# Supplementary figures and images for: Deep crypt secretory cells shape region-specific mucin glycosylation patterns in the mouse colon
Source: PLoS One. 2025 Jul 8;20(7):e0326157. doi: 10.1371/journal.pone.0326157 (PMC12237016; doi:10.1371/journal.pone.0326157)

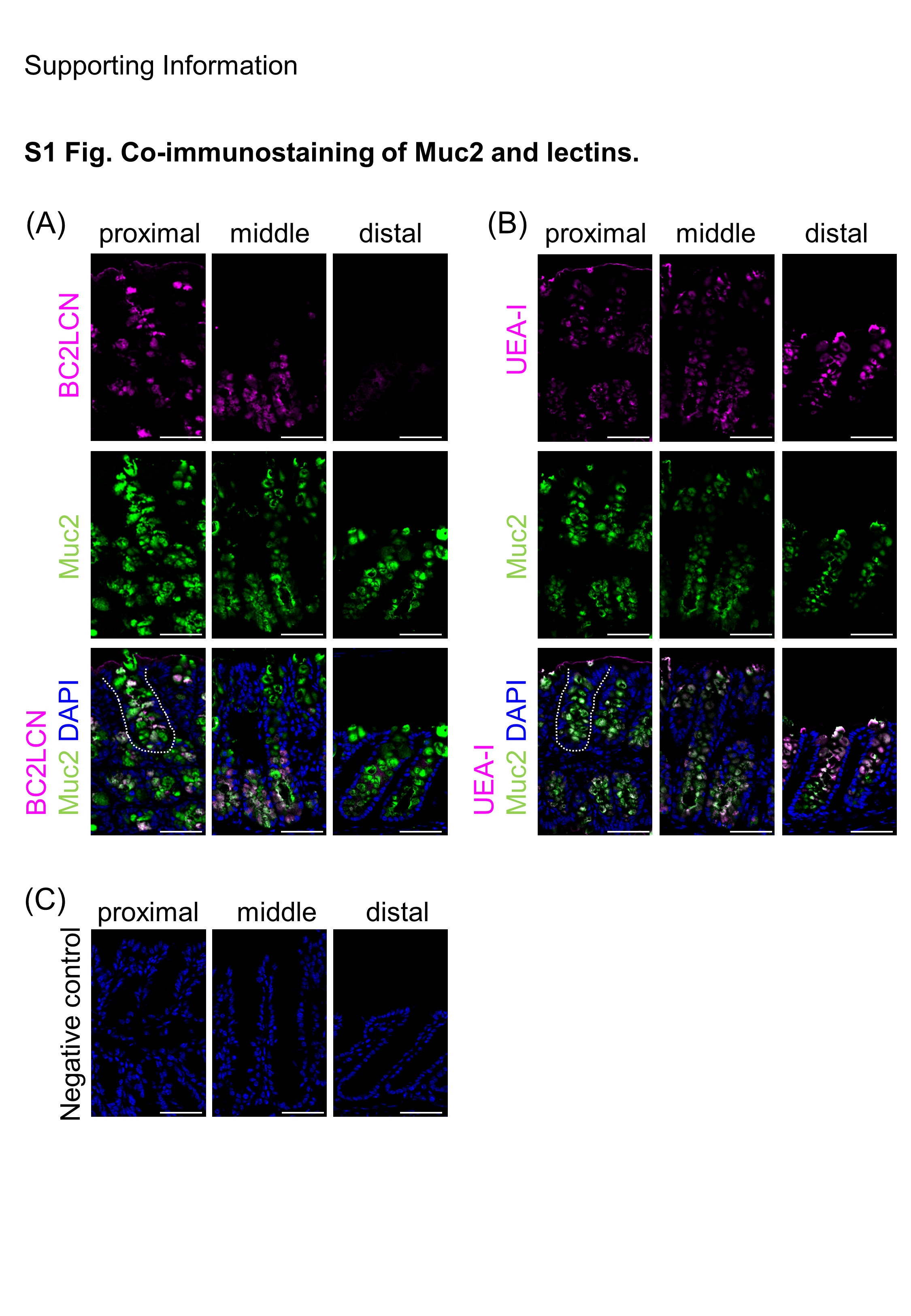

Supplement: S1 Fig — Murine colonic sections were stained with biotinylated BC2LCN or UEA-I (magenta) and anti-Muc2 antibody (green) following the procedures described in Materials and Methods, except that Alexa Fluor 488-conjugated donkey anti-rabbit IgG(H&L) polyclonal antibody (1:1200; ab150065, Abcam) was used as the secondary antibody for anti-Muc2 antibody. Nuclei were stained with DAPI (blue). Scale bar = 50 µm. Representative images from three independent experiments for each colonic region are shown. For clarity, representative crypts are marked with dotted lines. (A) Co-immunostaining of BC2LCN and Muc2. BC2LCN reactivity was confined to the middle to lower regions of the crypts, and co-localized with Muc2 signals. (B) Co-immunostaining of UEA-I and Muc2. UEA-I reactivity was localized to secretory granules and overlapped with Muc2 signals, including those in the middle to lower regions of the crypts in the proximal and middle colon. (C) Negative control: Staining performed without biotinylated lectins and the anti-Muc2 antibody. No significant signal was detected. (TIF) [file pone.0326157.s002.tif]
