# Supplementary material for: Deep crypt secretory cells shape region-specific mucin glycosylation patterns in the mouse colon
Source: PLoS One. 2025 Jul 8;20(7):e0326157. doi: 10.1371/journal.pone.0326157 (PMC12237016; doi:10.1371/journal.pone.0326157)
Supplement: Attachment — Submitted filename: Response to Reviewers_Sugahara et al.docx [file pone.0326157.s004.docx]

**Responses to Reviewers**

*Initial submission #: PONE-D-25-10344*

Dear Academic Editor and Reviewers,

We would like to sincerely thank the Academic Editor and Reviewers for their careful evaluation of our manuscript and for providing thoughtful and constructive comments to improve its clarity and quality.

In response, we have revised the manuscript accordingly. Below, we provide point-by-point responses to all comments from the Academic Editor and Reviewers. A summary of the changes made is as follows:

- All revisions in the manuscript are highlighted in blue.

- Newly generated Supporting Information files have been added:

S1 Data (XLSX)

S1 Fig (TIF)

- Reference numbering has been corrected:

(i) Three new references (#42, #48, and #49) have been added.

(ii) The original references #42–49 have been renumbered accordingly.

(iii) A citation error in the original manuscript at line 611 has been corrected: Nyström et al. is now properly cited as reference #41 (previously #40).

- These changes do not affect the interpretation of the results or the overall conclusions of the study.

We believe that the revised manuscript fully addresses the concerns raised, and we respectfully submit it for your further consideration.

Sincerely,

Daisuke Sugahara

(on behalf of all authors)

**Response to Academic Editor**

**General Comment**

The topic presented is both interesting and relevant, as the study addresses an important aspect of colon health: the glycosylation of mucins. This research identifies a new key player, DCS cells, which could have significant implications for our understanding of mucin biology and their role in colon health. By exploring the functions and mechanisms of these cells, the study opens new avenues for research that could lead to innovative therapeutic strategies and a deeper understanding of the complexities of mucin interactions within the colonic environment. This contribution is fundamental for advancing our knowledge and addressing related health issues.

**Response**

We appreciate the Editor’s thoughtful and encouraging comments on the significance of our study. We are pleased that the potential relevance of DCS cells to mucin biology and colon health was recognized, and we value your perspective on how this work may contribute to a deeper understanding of region-specific glycosylation and mucosal function. The editor’s feedback was very helpful in clarifying the broader implications of our findings in the revised manuscript.

**Comment (1)**

Has the expression of DCS been observed in other vertebrates (e.g., Balestra)? A comparison could be made. This could serve as a valuable starting point for further research to investigate the mechanisms regulating glycosylation among different vertebrate species. It would be possible to build on the data already published and integrate them into the text. Does DCS expression change under a variety of experimental conditions? Recent literature regarding alterations in the binding of glycosidic residues in the colons of mice fed a high-fat diet could be included, along with conducting an analysis of DCS expression.

**Response**

We sincerely appreciate the valuable comments. We understand that the comments mainly concern the following two points:

(i) Whether the presence of DCS cells has been observed in other vertebrate species?

(ii) Whether DCS cell distribution or expression changes under different experimental conditions?

Below, we provide our responses to each point.

(i)	We appreciate the insightful suggestion on the presence of DCS cells in other vertebrates, which is relevant to potential for the presence of a similar mechanism regulating mucin glycosylation across species. To date, there have been no reports directly indicating the presence of DCS cells or their equivalents in vertebrates other than mice and rats. DCS cells are considered counterparts analogous to the Paneth cells in the small intestine. While Paneth cells exhibit differences in morphology and distribution among animal species, they are known to exist across a broad range of mammals, including pigs, horses, rabbits, and cattle, as well as in chickens (reviewed in Cui C et al. *J Anim Sci Biotechnol*. 2023;14(1):118.). Although their apparent characteristics may differ from those of mice and rats, it is plausible that DCS cells, or analogous cell types, are also present in a wide variety of mammals. Among these, the spiral colon of pigs may be the most promising candidate. In a study by Lin SJ et al. (*Front Cell Infect Microbiol*. 2023;12:1042815.), lectin staining of pig colon tissue suggested the presence of Muc2-producing cells with distinct mucin glycosylation profiles at the lower parts of the crypts, resembling the DCS cells described in our current study.

Even more important from the perspective of maintaining human health and preventing disease, a recent study by Bustamante-Madrid P et al. (*Cell Death Dis*. 2024;15(4):301.) suggests the existence of DCS-like cells in health human colon. As we also mention in our reply to Reviewer#2’s comment (4), their study further indicated that Notch singling may be involved in the differentiation of these cells, consistent with previous findings in mice. However, further studies are needed to determine whether these DCS-like cells in pigs and humans are functionally equivalent to mouse DCS cells.

Considering the potential significance of DCS-like cells across species, we have added a new subsection to the *Discussion* section entitled “*Conservation of DCS-like cells across species*” (Line 660-685). In this new section, we cited their work and incorporate relevant descriptions (Line 673 and 679). Accordingly, the subsection “*Potential implications of human DCS cells in colonic diseases*” has been shortened, as some of its content was moved to the new subsection, and minor revisions have also been made to improve clarity and flow.

(ii)	We also appreciate the Editor’s suggestion regarding the inclusion of a recent study that investigates changes in glycan expression patterns under different experimental conditions such as high-fat diet. As the Editor points out, we also consider that the study by Mastridonato M et al. (*Histochem Cell Biol*. 2014;142(4):449-59.) indicates changes in DCS cell distribution under a particular experimental condition in the mouse colon. Their histochemical analysis revealed the presence of mucin-producing cells with distinct mucin glycosylation profiles located at the lower parts of the crypts. Although Mastridonato et al. did not specifically identify these cell types, we believe that these cells correspond to DCS cells. Notably, their results showed that the crypt regions occupied by these distinctive cells expanded in mice fed a high-fat diet, suggesting that the distribution (abundance) of DCS cells changes in response to dietary components.

Their study also indicated that mucin-producing cells in the proximal colon, likely including DCS cells, produce acidic glycans. This observation differs from our findings and those of Bergstrom K et al. (*Science*. 2020;370(6515):467-72.). Although the reason for this discrepancy remain unclear, their results may suggest that this cell population is responsive to dietary or environmental cues. Accordingly, we have therefore cited this study and briefly discussed it in the *Discussion* section of the revised manuscript (Line 649-659).

Additionally, the experimental results reported by Lin et al., cited above, may also suggest that the area occupied by DCS-like cells expands in response to microbial infection in the pig colon. These findings, together with those of Mastridonato et al., support our notion that DCS cells play crucial and unique roles in mucin production and the maintenance of colonic health. However, as we are not sufficiently familiar with the histological characteristics of the porcine large intestine, and since alternative interpretations were presented in their article, we have opted not to include this point in the revised manuscript. We nevertheless appreciate the opportunity to consider this perspective.

**Response to Reviewer#1**

**General Comment**

My opinion is that the findings in this original research article fit the scope of PLOS One and are scientifically sound and novel.

**Response**

We sincerely appreciate the reviewer’s positive evaluation, especially regarding the scientific soundness and novelty of our findings.

**Comment (1)**

However, to my knowledge Welch’s t-test assumes normal distribution of data but can be valid with a symmetric, non-skewed, sample size of more than 50 samples (https://doi.org/10.1177/0004563221992088). How was the data tested for normal distribution, or did it meet other criteria for Welch’s t-test to be used that I’m not aware of?

Can you supply the data underlying the quantitative analysis of the DCS cell distribution as supplementary information or deposited to a clearly stated public repository to meet the PLOS One data availability requirements?

**Response**

We appreciate the reviewer's comment on this point. In response to the reviewer’s suggestion, we have added the numerical data underlying the quantitative analysis of DCS cell distribution (Figs 1C and 1D) as Supporting Information (S1 Data). We have also referred to this addition in the revised manuscript (Line 254-255 and 270).

We believe that our data meet the assumptions for Welch’s *t*-test, as outlined in the literature cited in the reviewer’s comment (West RM. *Ann Clin Biochem*. 2021;58(4):267-69.). It has been suggested that if the mean of a dataset is more than twice its standard deviation, the distribution can be considered approximately normal or not strongly skewed. As shown in the table below (also included in S1 Data for transparency), the mean exceeded twice the standard deviation in all colonic regions, suggesting relatively low skewness in our data.

| Colonic region | Sample size (n) | Mean of DCS cell abundance (%) | Standard deviation (SD) | 2 x  SD | Mean > 2 x SD? | Normal distribution? |
| --- | --- | --- | --- | --- | --- | --- |
| Proximal | 68 | 67.3 | 11.6 | 23.1 | Yes | Yes/approximately |
| Anterior Middle | 42 | 63.8 | 8.4 | 16.9 | Yes | Yes/approximately |
| Posterior Middle | 55 | 57.0 | 7.9 | 15.7 | Yes | Yes/approximately |
| Distal | 109 | 37.3 | 13.0 | 25.9 | Yes | Yes/approximately |

Another important assumption for Welch’s *t*-test is that the sample size is sufficiently large; in practice, a sample size of n > 50 is often considered adequate. In our dataset, the smallest group size was n = 42 (from the anterior region of the middle colon), which is not substantially smaller than this threshold. Therefore, we consider the sample sizes in our study sufficiently large to justify the use of Welch’s *t* -test.

**Response to Reviewer#2**

**General Comment**

This is a nicely written manuscript reporting the gradient distribution of DCS cells along the colon and the underlying role of region specific glycosylation in colon health. The authors elaborate on previously reported research to determine correlation of Core3 glycosylation and proximal mucins, and provide scientific evidence linking it to DCS cell distribution. I have a few minor comments and questions as follows-

**Response**

We truly appreciate the reviewer’s kind and thoughtful comments highlighting both the significance of our findings and the quality of our manuscript. We are encouraged by this positive evaluation and have carefully addressed the comments and questions raised.

**Comment (1)**

Since this work is heavily relying on immunostaining, could the authors discuss if an isotope control was included in addition to the negative control, to confirm specificity of primary antibody? This is specifically, in reference to figure 4 (showing the UEA-1 reactivity in the in situ PLA).

**Response**

We thank the reviewer for raising the importance of including an isotype control to confirm the specificity of antibody-based staining. As for conventional immunostaining experiments (Figs 1, 2, and 3), we included appropriate negative controls by omitting the primary antibodies and lectins to confirm the specificity of the signals. Although we did not perform isotype control staining for each primary antibody used in this study, the observed staining patterns were highly consistent with previously reported or expected expression profiles.

As for PLA, to facilitate the interpretation of PLA controls, we additionally performed co-immunostaining using the same probe pairs (i.e., lectins and anti-Muc2 antibody) employed in the PLA experiments. These data have been included as Supporting Information (S1 Fig). Accordingly, we have referred to this addition in the manuscript (Line 461-463) and added some sentences (Line 474-483 and 525-527). We have also referred to S1 Fig in the relevant sentence (Line 607) to improve clarity.

To confirm the specificity of the anti-Muc2 antibody and to validate the lectin-based PLA system, we included two types of controls: (i) a positive control using BC2LCN lectin and anti-Muc2 antibody, which resulted in a PLA signal spatially consistent with the merged co-immunostaining signal of the same probe pair (S1A Fig); and (ii) a negative control omitting both primary probes (lectin and antibody), which produced no significant PLA signal (Fig 4C). These results collectively support the specificity of the antibody and the validity of the signals observed in lectin-based PLA.

Consequently, if any non-specific PLA signal were to be present in the UEA-I–based PLA (Fig 4B), it would most likely stem from the UEA-I itself. Regarding the reviewer’s suggestion of using an isotype control antibody in combination with UEA-I lectin, we believe that such an experiment would not effectively address the potential for nonspecific signals arising from the lectin probe. UEA-I is a lectin, not an antibody, and thus has no corresponding isotype control. In this context, omitting the lectin would be serve as a more practical and informative negative control. Moreover, the reactivity of UEA-I observed in co-immunostaining (Fig 2E and S1B Fig) was localized in secretory granules, consistent with previous reports (Imai Y et al. *Infect Immun*. 2003;71(2):985-90; Liquori GE et al. *Acta Histochem*. 2012;114(7):723-32.). Thus, we consider the likelihood of nonspecific UEA-I–mediated PLA signal to be minimal.

There is also a possibility that PLA signals may arise from nonspecific interactions between the antibody and UEA-I, a concern inherent to PLA, which depends on the spatial proximity of the probes. If such nonspecific interactions occurred in the UEA-I–based PLA, signals would be expected across all secretory granules, where both probes are present. However, as shown in Fig 4B, no PLA signals were detected in DCS cells in the proximal and middle colon, despite the presence of both Muc2 and UEA-I reactivity (S1B Fig). This observation suggests that nonspecific interactions between the probes are minimal. Taken together, we believe that our study includes sufficient validation of the specificity and signal reliability in the lectin-based PLA system.

Alternatively, had such clear validation not been achieved, a competition assay using a monosaccharide—such as fucose, which inhibits UEA-I binging—and enzymatic digestion—such as α1,2-fucosidase digestion treatment—could have been employed to assess specificity in lectin-based PLA. However, in light of the robust positive and negative controls already in place, we consider further controls to be unnecessary in the present study.

**Comment (2)**

I am also curious why Adobe photoshop was used for image analysis, instead of other open source software available for scientific image analysis.

**Response**

We thank the reviewer for the comment. Adobe Photoshop was used solely for image presentation purposes. Specifically, we applied levels adjustment uniformly across each image to enhance the visibility of fluorescence signals and to ensure consistent contrast across figure panels. No measurements, cell counting, or other quantitative analyses were performed using Adobe Photoshop. We have revised the manuscript in the *Materials and Methods* section to clarify this point and included the following descriptions;

Line 160-162 (*Quantitative analysis of DCS cell distribution*)

The number of marker-positive cells was manually counted by visual inspection of the images, using consistent criteria throughout the study.

Line 199-202 *(Fluorescent imaging using a confocal microscope*)

Fluorescence images were processed using Adobe Photoshop (San Jose, CA) to adjust brightness and contrast. Identical Level Adjustment settings were uniformly applied to all images within each figure panel. These modifications were made exclusively for presentation purposes and did not affect any data analysis.

We appreciate the reviewer’s comment, which provided an opportunity to clarify our image processing procedures and to emphasize our commitment to scientific transparency.

**Comment (3)**

Other than mucin production, are there any more functional differences expected in the DCS cells distributed along the proximal, middle and distal colon?

**Response**

We appreciate this comment by the reviewer. Our current findings reveal regional differences in mucin-glycan production by DCS cells, as exemplified by minimal lectin reactivity (Fig 2) and the absence of C3GnT expression (Fig 3). These distinctive cellular properties of the distal DCS cells may influence other DCS cell functions, underscoring the potential for region-specific specialization of DCS cell functions. Although niche functions regulating stem cell activity are among the best-recognized roles of DCS cells, most studies investigating DCS functions have focused primarily on a particular region, namely the distal colon, often considering it representative of the entire colonic region. In this context, it remains uncertain whether their roles are uniformly maintained across the entire length of the colon. If not, the niche functions in the proximal and middle colon may instead be carried out by other cell types, or a specific subset of DCS cells, implying regional differences in the mechanisms regulating stem cell activity along the colon. Accordingly, clarifying potential regional distinctions in DCS cell functions will provide important insights into the previously underappreciated complexity of their biology and the mechanisms underlying the maintenance of homeostasis in the colon.

**Comment (4)**

Line 620-624 discuss the variations in the Notch and Wnt signaling pathways (presumable originating from Lgr+ stem cells) that could drive the differences in DCS distribution and glycosylation. Since Notch and Wnt are highly conserved across species, can the authors comment if this has been studied in human organoid/stem cell models or if they plan to? This could help hypothesize similar regional glycosylation differences and help address the limitations (in line 640-641) of extending this work to human DCSs.

**Response**

We are grateful for this insightful and constructive suggestion. Exploring conserved signaling pathways such as Notch and Wnt in human organoid or stem cell models indeed offers a promising avenue to overcome current limitations in extending our findings to human DCS cells. We agree that this perspective significantly enhances the relevance and future potential of our study.

While previous studies have shown that Notch and Wnt signaling can promote differentiation into “goblet cells” (as conventionally defined) in human colonic organoids, the differentiation of DCS cells in such models had not been examined until recently. Notably, a study by Bustamante-Madrid P et al. (*Cell Death Dis*. 2024;15(4):301.) provided supportive evidence for the existence of DCS-like cells in human colon and further suggested that Notch signaling may regulate their differentiation using human colonic organoids. We have cited this study and included some sentences in the *Discussion* section of the revised manuscript (Line 673-678 and 682-685).
